# Long-term seizure outcome after epilepsy surgery of neuroglial tumors

**DOI:** 10.3389/fneur.2024.1384494

**Published:** 2024-05-23

**Authors:** Attila Rácz, Philipp Müller, Albert Becker, Nico Hoffmann, Theodor Rüber, Valeri Borger, Hartmut Vatter, Rainer Surges, Christian E. Elger

**Affiliations:** ^1^Department of Epileptology, University Hospital Bonn, Bonn, Germany; ^2^Department of Neuropathology, University Hospital Bonn, Bonn, Germany; ^3^Department of Neuroradiology, University Hospital Bonn, Bonn, Germany; ^4^Department of Neurosurgery, University Hospital Bonn, Bonn, Germany

**Keywords:** neuroglial tumor, ganglioglioma, dysembryoplastic neuroepithelial tumor, DNET, epilepsy surgery, outcome, hippocampal sclerosis (HS)

## Abstract

**Purpose:**

Neuroglial tumors are frequently associated with pharmacorefractory epilepsies. However, comprehensive knowledge about long-term outcomes after epilepsy surgery and the main prognostic factors for outcome is still limited. We sought to evaluate long-term outcomes and potential influencing factors in a large cohort of patients who underwent surgery for neuroglial tumors in a single-center setting.

**Methods:**

The study analyzed the outcomes of 107 patients who underwent epilepsy surgery for neuroglial tumors between 2001 and 2020 at the Department of Epileptology, University Hospital Bonn, in Germany. The outcomes were evaluated using Engel classification. Differences in outcome related to potential prognostic factors were examined using the Chi2-test, Fisher’s exact test and sign test. Additionally, stepwise logistic regression analysis was employed to identify independent prognostic factors.

**Results:**

Complete seizure freedom (Engel Class IA) was achieved in 75% of the operated patients at 12 months, and 56% at the last follow-up visit (70.4 ± 6.2 months, median: 40 months). Completeness of resection was a crucial factor for both 12-month follow-up outcomes and the longest available outcomes, whereas lobar tumor localization, histology (ganglioglioma vs. dysembryoplastic neuroepithelial tumor), history of bilateral tonic–clonic seizures prior to surgery, invasive diagnostics, side of surgery (dominant vs. non-dominant hemisphere), age at epilepsy onset, age at surgery, and epilepsy duration did not consistently impact postsurgical outcomes. Among temporal lobe surgeries, patients who underwent lesionectomy and lesionectomy, including hippocampal resection, demonstrated similar outcomes.

**Conclusion:**

Neuroglial tumors present as excellent surgical substrates in treating structural epilepsy. To achieve an optimal postsurgical outcome, a complete lesion resection should be pursued whenever possible.

## Introduction

Neuroglial tumors frequently contribute to pharmacologically challenging epilepsies, especially in the temporal lobe (1–5). These lesions are commonly believed to be congenital or acquired very early in life (5), with most cases displaying either no significant growth or a slow growing tendency without malignant transformation (6). Traditionally, ganglioglioma (GG) and dysembryoplastic neuroepithelial tumors (DNETs) have been considered the primary representatives of this lesion class, as reflected in the WHO classification of brain tumors (7). However, recent advancements in molecular biology have introduced new entities, such as polymorphous low-grade neuroepithelial tumors of the young (PLNTY) (8) and multinodular and vacuolating neuronal tumors (MVNT) (9), which strongly resemble GG or gangliocytoma. These lesions are predominantly located in the temporal lobe (5, 10), with frequent involvement of temporomesial structures (10). Conjunctional hippocampal sclerosis (4, 6) and a certain degree of disorganization in the cortical lamellar architecture are not uncommon findings either. Several studies emphasize the importance of these lesions as epileptogenic lesions, which are the second most frequently identified histopathology in resected specimens of epilepsy surgery patients, both in children and adults (1). Evidence also supports favorable postsurgical outcomes after epilepsy surgery (10–16). Although GG and DNET frequently exhibit distinct genetic alterations, certain immunohistochemical features, such as expression of CD34, can be found in both (6). GG frequently displays BRAF V600E mutations (17–20), whereas DNET, a histological class with further subtypes (6, 21), shows frequent mutations in FGFR1 (17, 22). Neuroglial tumors can also be associated with FCD (3, 4, 18, 23). Stereo-EEG (SEEG) investigations have provided evidence for an intrinsic epileptogenicity of neuroglial tumors (3), suggesting that the way they induce seizures may differ from those of “conventional tumors.”

Studies evaluating the postsurgical outcomes with neuroglial tumors are diverse, varying in terms of the number of included patients, duration of outcomes, and examined histopathologic entities. Predictors of favorable postsurgical outcomes also vary among different studies. A pooled analysis of 910 patients stemming from 39 studies identified gross tumor resection and a short epilepsy duration (less than 1 year) as positive predictors, whereas the history of bilateral tonic–clonic seizures was identified as a negative predictor (16). Additionally, in this study, the resection of the hippocampus along with the neuroglial tumors in temporal lobe epilepsies was associated with improved outcomes (16). This finding was also corroborated by a recent study involving 35 patients (24). However, other studies with smaller sample size also documented excellent outcomes with amygdala resection alone, without involving the hippocampus in the resection scheme (25).

In this study, we aimed to expand our knowledge of long-term outcomes after epilepsy surgery with neuroglial tumors and identify potential predictors in a single-center setting. Furthermore, our objective was to analyze the impact of hippocampal resection on postsurgical outcomes in temporal lobe epilepsy within our patient cohort.

## Patients and methods

We conducted a retrospective study which included patients who underwent epilepsy surgery for neuroglial tumors at our center (Department of Epileptology, University Hospital Bonn, Germany) between 2001 and 2020. These patients underwent a comprehensive presurgical evaluation including a brain MRI, video EEG monitoring with seizure recording, neuropsychological tests, and, in certain cases (14 patients), invasive EEG diagnostics. Histopathologic analysis of surgical specimens was performed in the Department of Neuropathology of the University Hospital Bonn, Germany. To maintain a more homogeneous patient cohort, those patients who had previously undergone surgery in another department and required a second surgery in our center were excluded from our evaluation. This exclusion was based on the principle that second surgeries might be associated with somewhat worse outcomes than initial surgeries (26, 27). Previous biopsies of the lesions were not an exclusion criterion. Additionally, patients exhibiting potential dual pathologies other than hippocampal sclerosis, and cases with anaplastic GG were also excluded. Only patients with a minimum of 10 months [extrapolated as a 12-month follow-up (FU)] were considered eligible for the study. Patients not displaying pharmacorefractory epilepsy before surgery were also not enrolled. After these considerations, 107 patients fulfilled the criteria for the primary investigation. Data were extracted and collected from clinical records and phone interviews. Additional patient characteristics are presented in Table 1. Outcomes were classified using Engel’s classification system (28). Statistical evaluation was performed using the Matlab statistics toolbox. Outcome differences between the 12-month FU and the longest available FU were evaluated with a sign test. In patients with two surgeries, only the results from the first surgery were considered in the primary analysis, while outcomes following the second surgery were reported separately. We tested a series of potential prognostic factors for postsurgical outcomes in a univariate analysis, applying Chi2-statistics and Fisher’s exact tests. In these analyses, we assessed excellent (Engel Class I) vs. non-excellent (Engel Classes II–IV) and favorable (Engel Classes I–II) vs. poor (Classes III and IV) outcome distributions. Subsequently, we evaluated the impact of these factors, as well as age at epilepsy onset, age at surgery, and epilepsy duration (as continuous variables) on seizure outcome (excellent vs. non-excellent and favorable vs. poor outcome) using stepwise logistic regression analysis. Certain aspects and outcomes analyzed according to different questions in overlapping patient populations have been published elsewhere (29–31). The study was conducted with the respective approval of the local ethics committee (Ethikkommission an der Medizinischen Fakultät der Rheinischen Friedrich-Wilhelms-Universität Bonn, No. 126/19). Informed consent was required for phone interviews only.

**Table 1:** Patient characteristics for operations with neuroglial tumors.

Localization	GG^*^	DNET	Overall
Frontal	4 (4.5%)	5 (26.3%)	**9 (8.4%)**
Temporal	65 (73.9%)	9 (47.4%)	**74 (69.2%)**
Parietal	4 (4.5%)	2 (10.5%)	**6 (5.6%)**
Occipital	1 (1.1%)	0 (0%)	**1 (0.9%)**
Insular	5 (5.7%)	0 (0%)	**5 (4.7%)**
Multilobar	9 (10.2%)	3 (15.8%)	**12 (11.2%)**
Total	**88 (100%)**	**19 (%100)**	**107**
FTBTCS	FTBTCS	no FTBTCS	Unclear
Number of patients	67 (62.6%)	30 (28%)	10 (9.3%)
Gender	Male	Female	Overall
Number of patients	54 (50.5%)	53 (49.5%)	**107**
Resection	Complete	Incomplete	Unclear
Number of patients	76 (71%)	13 (12.1%)	18 (16.8%)
Invasive diagnostics	Yes	No	Overall
Number of patients	14 (13.1%)	93 (86.9%)	**107**
Side of surgery	Left	Right	Overall
Number of patients	48 (44.9%)	59 (55.1%)	**107**
Side of surgery	Dominant	Non-dominant	Unclear
Number of patients	36 (33.6%)	47 (43.9%)	24 (22.4%)
Age at epilepsy onset (mean ± SD)		14.71 ± 10.66 years (sem = 1.04, median = 13)	
Age at surgery (mean ± SD)		29.26 ± 15.96 years (sem = 1.54, median = 27.75)	
Duration of epilepsy (mean ± SD)		14.53 ± 14.17 years (sem = 1.38, median = 9.35)	

FTBTCS, focal to bilateral tonic–clonic seizure. GG^*^, this group also included five cases with PLNTY und one case with MVNT. For clarification, see the Results section. For two patients, the age at epilepsy onset and epilepsy duration could not be determined precisely.

SD, standard deviation; sem, standard error of the mean.

## Results

**Figure 1:**
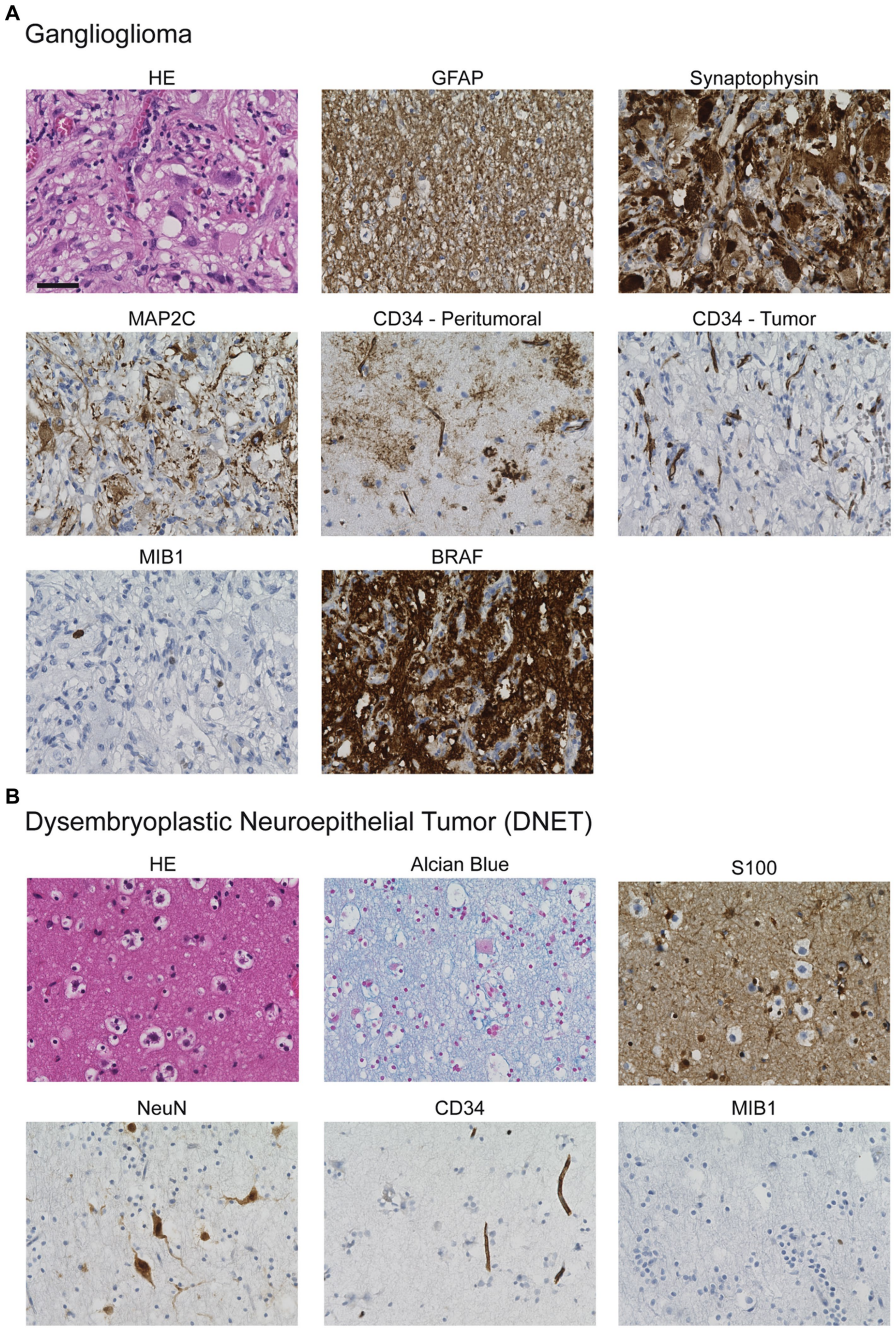
Histological appearance of **(A)** GGs and **(B)** DNETs. **(A)** HE-staining reveals the biphasic composition of GGs with dysmorphic neurons embedded in a glial matrix. The neuronal component typically shows positivity for synaptophysin- and MAP2C-staining whereas the glial component is characterized by expression of GFAP. Additionally, CD34 is frequently expressed in the peritumoral and tumoral area. Typically, proliferative activity is low as indicated by MIB1-staining. BRAF-staining may detect the typical BRAF V600E mutation. **(B)** HE-staining of DNET shows the glioneuronal element with glial cells and floating neurons embedded in an alcianophilic matrix. The glial component can be detected by S100-expression, whereas the neuronal component expresses NeuN. CD34 may be expressed, here it is restricted to endothelial cells. The proliferative activity is low. Scale bar: 50 μm.

From the patients who underwent epilepsy surgery in our center for medically difficult-to-treat epilepsies with neuroglial tumors between 2001 and 2020, 107 met the inclusion criteria. The basic characteristics of this patient group are outlined in Table 1. Thirty-two patients (30%) underwent surgery before the age of 18, while 75 patients (70%) were operated on after turning 18 years of age. The typical histopathologic features of GG and DNET are illustrated in Figure 1.

**Figure 2:**
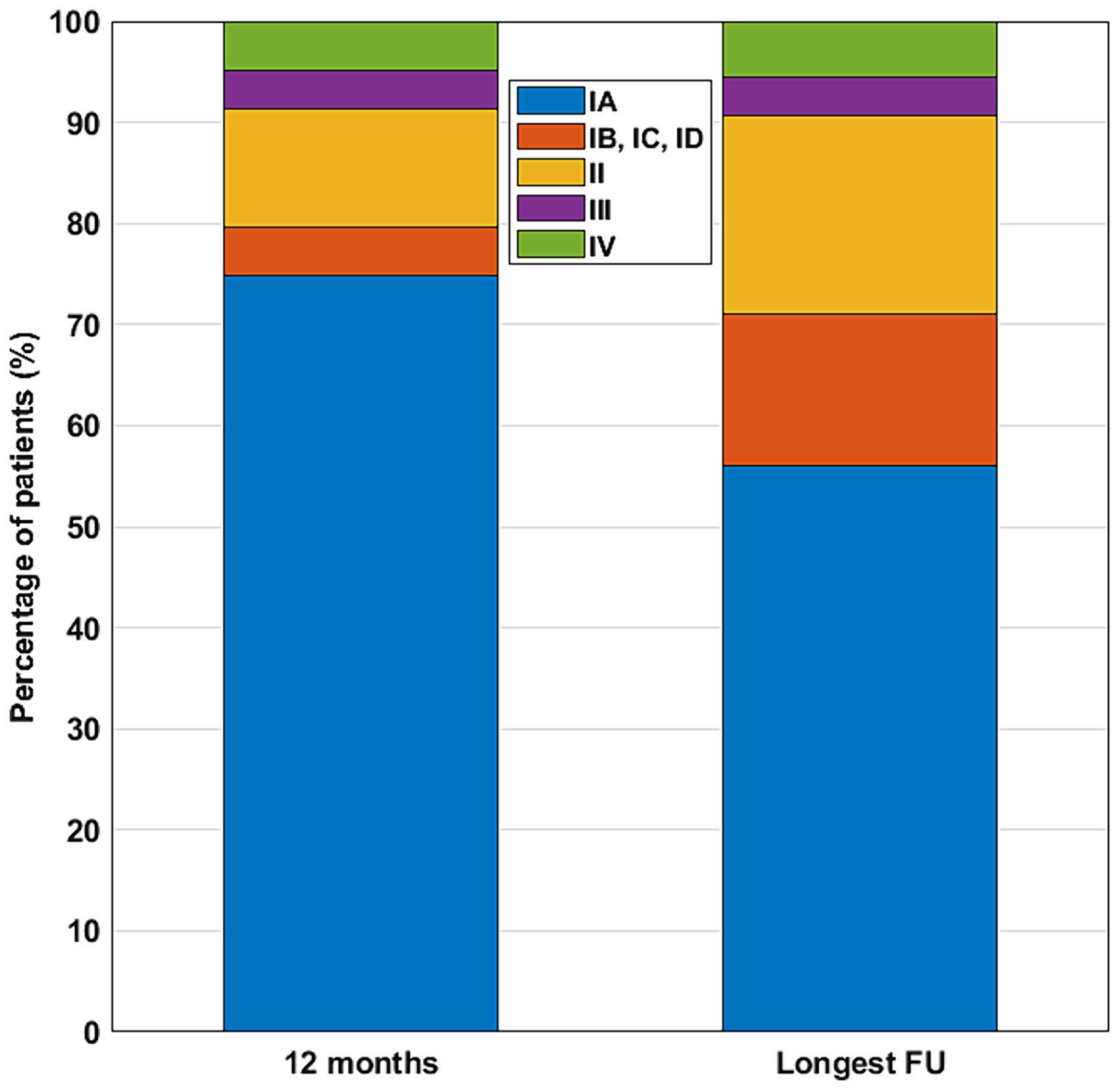
Postsurgical outcomes at the 12-month and at the longest available FU. Color code for outcome classes: IA—blue; IB, IC, ID—red; II—yellow; III—purple; IV—green.

At the 12-month FU, the outcomes of four patients were not precisely determined (only available at the latest FUs). At the 12-month FU, 77 patients (74.8%) were completely seizure free (Engel Class IA), and 82 patients (79.6%) achieved an excellent outcome (Engel Class I; Figure 2). At the longest available FU (70.4 ± 6.2 months, mean and standard error of the mean; median 40), 60 patients (56.1%) were completely seizure free, and 76 (71%) had an excellent (Engel Class I) outcome (Figure 2). Outcomes were significantly worse at the last available FU, compared to the 12-month FU (*p* < 0.01, sign test; Figure 2). By the time of the 12-month FU, 99 patients were still taking antiseizure pharmacotherapy, and five discontinued pharmacotherapy (in three cases, we did not have clear information on drug use). At the longest available FU, 79 patients were still taking antiseizure medication, 28 had already stopped taking it, and 35 could switch from an antiseizure combination treatment to a monotherapy.

From the reported patient group, five patients also underwent a second surgery (four of them with incomplete resections at the first surgery). Among these patients, four showed improvement (in one of them, the Engel class improved from IV to II; in another, the Engel class changed from III to II; in a further one, the Engel class changed from II to IC; and in the last one, from IV to IB), while one did not improve (Engel Class II).

Out of the 107 patients with neuroglial tumors, 82 were originally classified as GG, and 19 as DNET. Among those patients who underwent surgery after 2016, we identified five cases with a histopathologic diagnosis of PLNTY, and one case with MVNT. Since these new histopathologic entities show characteristics similar to GG, and most probably would have been previously diagnosed as GG, we included them in the GG group as well. Patients operated on with GG and DNET did not present significantly different outcome measures either at the 12-month FU, or at the longest FU. For outcome classes and *p*-values of Chi2-tests and Fisher’s exact tests, respectively, see Table 2.

**Table 2:** Possible predictors of postsurgical outcome—univariate analysis.

Predictors		Histology				Localization				Extent of resection			
		12 months		Last FU		12 months		Last FU		12 months		Last FU	
Engel I vs. Engel II-III-IV	*p*-value	0.76^b^		0.16^a^		0.19^a^		0.26^a^		***<0.01*** ^***a***^		***<0.001*** ^***b***^	
Engel I-II vs. Engel III-IV	*p*-value	0.65^b^		1.00^b^		0.13^b^		0.07^b^		***<0.05*** ^***b***^		***<0.001*** ^***b***^	
		**GG**	**DNET**	**GG**	**DNET**	**Tem.**	**Nont.**	**Tem.**	**Nont.**	**Com.**	**Inc.**	**Com.**	**Inc.**
Outcomes (Engel)	IA	75.29 (64)	72.22 (13)	56.82 (50)	52.63 (10)	77.46 (55)	68.75 (22)	58.11 (43)	51.52 (17)	80.82 (59)	30.77 (4)	65.79 (50)	7.69 (1)
Percentage (%) and number of patients	IB, IC, ID	4.71 (4)	5.56 (1)	17.05 (15)	5.26 (1)	5.63 (4)	3.13 (1)	16.22 (12)	12.12 (4)	2.74 (2)	15.38 (2)	11.84 (9)	15.38 (2)
	II	11.76 (10)	11.11 (2)	17.05 (15)	31.58 (6)	11.27 (8)	12.50 (4)	20.27 (15)	18.18 (6)	9.59 (7)	23.08 (3)	17.11 (13)	30.77 (4)
	III	4.71 (4)	0 (0)	4.55 (4)	0 (0)	2.82 (2)	6.25 (2)	1.35 (1)	9.09 (3)	5.48 (4)	0 (0)	2.63 (2)	15.38 (2)
	IV	3.53 (3)	11.11 (2)	4.55 (4)	10.53 (2)	2.82 (2)	9.38 (3)	4.05 (3)	9.09 (3)	1.37 (1)	30.77 (4)	2.63 (2)	30.77 (4)
Predictors		FTBTCS				Invasive DG				Side of surgery			
Engel I vs. Engel II-III-IV	*p*-value	0.50^a^		0.78^a^		0.73^b^		0.34^b^		0.68^a^		0.99^a^	
Engel I-II vs. Engel III-IV	*p*-value	0.72^b^		0.49^b^		1.00^b^		0.35^b^		1.00^b^		1.00^b^	
		**No**	**Yes**	**No**	**Yes**	**No**	**Yes**	**No**	**Yes**	**Dom.**	**Ndom.**	**Dom.**	**Ndom.**
Outcomes (Engel)	IA	75.86 (22)	73.44 (47)	63.33 (19)	52.24 (35)	74.16 (66)	78.57 (11)	55.91 (52)	57.14 (8)	68.57 (24)	76.60 (36)	55.56 (20)	59.57 (28)
Percentage (%) and number of patients	IB, IC, ID	6.90 (2)	3.13 (2)	6.67 (2)	14.93 (10)	4.49 (4)	7.14 (1)	12.90 (12)	28.57 (4)	8.57 (3)	4.26 (2)	16.67 (6)	12.77 (6)
	II	10.35 (3)	12.5 (8)	16.67 (5)	23.88 (16)	12.36 (11)	7.14 (1)	20.43 (19)	14.29 (2)	14.29 (5)	10.64 (5)	19.44 (7)	17.02 (8)
	III	3.45 (1)	4.69 (3)	6.67 (2)	2.99 (2)	3.37 (3)	7.14 (1)	4.30 (4)	0 (0)	2.86 (1)	2.13 (1)	2.78 (1)	2.13 (1)
	IV	3.45 (1)	6.25 (4)	6.67 (2)	5.97 (4)	5.62 (5)	0 (0)	6.45 (6)	0 (0)	5.71 (2)	6.38 (3)	5.56 (2)	8.51 (4)

FTBTCS, focal to bilateral tonic–clonic seizure; FU, follow-up; GG, Ganglioglioma; DNET, dysembryoplastic neuroepithelial tumor; Tem., temporal; Nont., non-temporal, multilobar or insular; Com., complete; Inc., incomplete; DG, diagnostics; Dom., dominant; Ndom., non-dominant. Results are displayed by p-values of Chi2-tests and Fisher’s exact tests comparing excellent with non-excellent (Engel Class I vs. Engel Classes II–IV); and favorable with poor outcomes (Engel Classes I–II vs. Engel Classes III–IV). Percentage and number of patients with respective outcomes are indicated accordingly. *P*-values from Chi2-test are indicated with ^a^, *p*-values from Fisher’s exact test are indicated with ^b^. Bold values indicate significant differences.

Most of the lesions (69.2%) were located in the temporal lobe; we investigated outcomes for lesions in temporal vs. extratemporal localizations (30.8%). Outcomes were not different for temporal and extratemporal lesion localizations, either at the 12-month FU or at the last available FU (Table 2).

**Figure 3:**
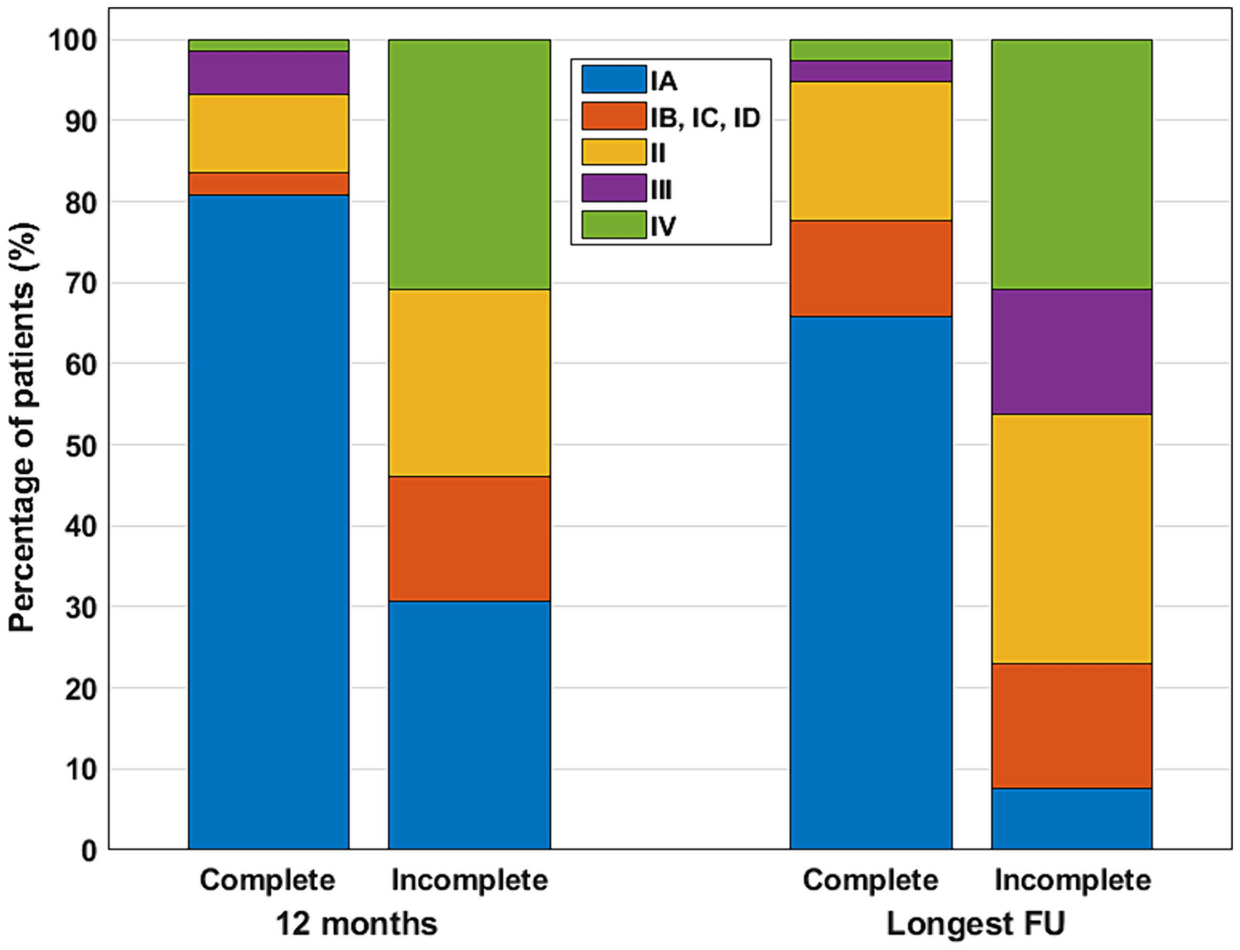
Postsurgical outcomes at the 12-month and at the longest available FU for patients with complete and incomplete lesion resection. Color code for outcome classes: IA—blue; IB, IC, ID—red; II—yellow; III—purple; IV—green.

Complete lesion resection was confirmed on postsurgical MRI images in 76 cases, while incomplete resection was observed in 13 cases. In 18 cases, the MRI findings were inconclusive regarding the completeness of resection, or discordant findings were noted in distinct postsurgical images, or no MRI images were available after surgery. The univariate analysis identified complete lesion resection as an important predictor for both 12-month FUs and the longest available FU outcomes (Table 2; Figure 3).

A history of bilateral tonic–clonic seizures prior to surgery has been thought to negatively influence postsurgical outcomes in patients with different histopathologies (12, 15, 16). Bilateral tonic–clonic seizures were reported in 68.6% of patients with temporal tumors and in 70.4% of patients with neuroglial tumors in extratemporal localizations (66.7% in frontal, 80% in parietal, 100% in occipital—though this was only one case—and 66.7% in multilobar, including insular, localizations; cases with unclear findings were not included). However, in the present cohort of patients with neuroglial tumors, a history of bilateral tonic–clonic seizures did not have a significant impact on postsurgical outcomes (either at the 12-month FU, or at the longest available FU, Table 2).

Invasive presurgical diagnostics (prior to surgery) were performed in 14 patients: with 12 patients undergoing the procedure during the period between 2001 and 2010 (from 66 surgeries), and two patients between 2011 and 2020 (from 41 surgeries). Although the frequency of invasive diagnostics was lower in the second period, the difference did not reach statistical significance (*p* = 0.07, Fisher’s exact test). Additionally, invasive diagnostics did not result in different outcomes compared to patients who did not undergo invasive monitoring (for results of the univariate analysis, see Table 2). Further information involving invasive monitoring and implantation schemes and strategies can be found in Table 3. Intraoperative electrocorticography (ECoG) was utilized in three cases during the period from 2001 to 2010.

**Table 3:** Invasive presurgical diagnostics in patients operated on with neuroglial tumors.

Lesion localization	Histop.	Impl. sides	Implantation scheme	Outcome 12 M	Outcome last FU
Left temporo-lateral	GG	One side	Subdural grid electrodes (left temporo-parietal)	IA	IC
Left temporo-lateral	GG	One side	Subdural strip and grid electrodes (grid left temporo-lateral, three strips left temporo-basal)	IA	IA
Left temporo-basal	GG	Both sides	Depth and subdural strip electrodes (hippocampal depth electrodes on both sides, strips temporo-lateral on both sides, two strips temporo-basal on the right, three strips temporo-basal on the left side)	IA	IA
Left temporo-mesial	GG	Both sides	Depth and subdural strip electrodes (hippocampal depth electrode on the left side, strips temporo-basal on both sides, left temporo-lateral and left fronto-lateral)	III	II
Right temporo-mesial	GG	Both sides	Depth and subdural strip electrodes (hippocampal depth electrodes on both sides, three strips fronto-mesial on both sides, one strip temporo-lateral on both sides)	IA	IA
Right temporo-mesial	GG	One side	Subdural strip electrodes (three strips right temporo-basal)	IA	IA
Right temporo-lateral	GG	One side	Subdural grid electrodes (right temporo-lateral)	IA	IB
Right temporo-mesial	GG	One side	Depth electrodes (one depth electrode right temporo-mesial)	IA	IA
Right temporo-lateral	GG	One side	Subdural grid electrodes (right temporo-lateral)	IA	IA
Left insular	GG	One side	Depth electrodes (two depth electrodes left insular)	IA	IA
Left fronto-parietal	GG	One side	Subdural strip electrodes (five interhemispheric strips on the left side)	IB	IB
Left parieto-occipital	GG	One side	Depth electrodes (four depth electrodes left temporal, one depth electrode left parietooccipital)	II	II
Right parieto-occipital	GG	One side	Depth electrodes (three depth electrodes right temporal, two depth electrodes right parieto-occipital)	IA	ID
Right temporo-occipital	GG	Both sides	Depth and subdural strip electrodes (one hippocampal depth electrode on the right side, five temporo-occipito-basal and temporo-occipito-lateral strips on the right side, two strips left temporo-basal)	IA	IA

Histop., Histopathology; Impl., Implantation; GG, Ganglioglioma; M, month; FU, follow-up.

Surgeries were performed on the left hemisphere in 48 cases, and on the right hemisphere in 59 cases. Further diagnostics to precisely determine hemispheric language dominance were carried out in 35 patients: intracarotid amobarbital test (Wada-test) was performed in nine cases, functional MRI was performed in 32 cases, functional transcranial Doppler sonography (fTCD) for language functions was performed in four patients. A combination of diagnostic tools was used in nine patients, with one additional case undergoing awake surgery with intraoperative speech monitoring. Among the patients who also underwent invasive diagnostics, precise demarcation of language areas was an indication factor in four cases. Surgery was performed on the dominant side (or presumably dominant, based on handedness) in 36 patients, and on the non-dominant (or presumably non-dominant) side in 47 patients. In the remaining cases, there was either insufficient information on hemispheric dominance or handedness, or the diagnostics provided confounding lateralizing information or hints at a bilateral speech representation, or hemispheric dominance was too early to determine (early childhood). Surgeries on dominant and non-dominant sides were associated with similar outcomes (both at the 12-month FU, and the longest available FU, as seen in Table 2). Incomplete resections were evenly distributed between the dominant and non-dominant hemispheres (five on the dominant, and eight on the non-dominant hemisphere, *p* = 0.99, Chi2-test). Interestingly, resections in extratemporal localizations on the dominant side were relatively infrequent (temporal lobe surgeries: 30 on the dominant, 27 on the non-dominant side; extratemporal surgeries: six on the dominant and 20 on the non-dominant side; *p* < 0.05, Chi^2^-test).

Subsequently, we tested the listed categorical variables (histopathology, lobar lesion localization, completeness of resection, bilateral tonic–clonic seizures, invasive diagnostics, lesion side) and additional potential predictors (age at epilepsy onset, age at epilepsy surgery, duration of epilepsy) with a stepwise logistic regression analysis. At the 12-month FU, a complete lesion resection was identified as the only independent predictor for an optimal postsurgical outcome (Table 4). At the longest FU, completeness of resection proved to be a significant factor for comparing excellent vs. non-excellent outcomes and favorable vs. poor outcomes. Additionally, the latest analysis (Engel Class I–II vs. III–IV) verified younger age at epilepsy onset as a further significant positive predictor (Table 4).

**Table 4:** Stepwise logistic regression analysis on possible predictors of postsurgical outcome.

	12-month FU		Last FU	
Outcome	Engel I vs. II–IV	Engel I–II vs. III–IV	Engel I vs. II–IV	Engel I–II vs. III–IV

Predictors	Extent of resection; *p* < 0.01 OR = 5.98 (1.59–22.56)^*^	Extent of resection; *p* < 0.05	Extent of resection; *p* < 0.001 OR = 11.52 (2.69–49.30)	Extent of resection; *p* < 0.01
		OR = 14.64 (1.80–119.14)		OR = 116.28 (5.15–2626.4)
		*Age at epilepsy onset*; *p = 0.06*		Age at epilepsy onset; *p* < 0.05
		*OR = 0.92 (0.84–1.00)*		OR = 0.87 (0.77–0.99)

FU, follow-up. OR, Odds ratio. 95% confidence intervals (in brackets) are indicated accordingly. Note that for continuous variables (in this case *Age at epilepsy onset*) odds ratios represent changes in 1 year increment. ^*^For the variable *Extent of Resection* reference is an incomplete resection.

### Temporal lobe surgeries

From the 74 neuroglial tumors with temporal lobe localization, 43 displayed at least a partial temporo-mesial affection, while the remaining 31 had temporo-lateral, temporo-basal or temporo-polar localization without temporo-mesial involvement. An amygdalohippocampectomy was performed in 39 patients (52.7% of temporal lobe surgeries), either in addition to the lesion removal, or intended directly to lesion removal for lesions located in the amygdala and/or hippocampus. The resection of the hippocampus did not significantly influence the 12-month FUs and long-term seizure outcomes in a univariate analysis compared to those patients who had temporal lobe surgery, but without hippocampal removal. For outcome distributions and statistics see Table 5. Six patients with temporo-mesial lesion affection did not undergo hippocampal resection initially; five of them reached excellent outcomes (Engel Class IA), and one achieved an Engel Class IV outcome. The latter patient underwent further resection, including the hippocampus, and achieved a long-term favorable (IC) outcome. In these six patients, the hippocampus did not exhibit any involvement or signal alteration on the MRI before surgery, despite the temporo-mesial involvement of the tumors. In another four patients with temporo-mesial neuroglial tumors and without hippocampal signal alteration, a hippocampal resection (in three cases hippocampus head) was performed due to the lesion’s vicinity to the hippocampus, or based on the results of the invasive diagnostics confirming hippocampal involvement in the seizure generation (long-term outcomes: two patients with Engel Class IA, one patient with Engel Class IB, and one patient with Engel Class II). Among those patients without temporo-mesial tumor involvement, the resection volume included the hippocampus only twice. One patient achieved complete seizure freedom after surgery, while the other achieved an excellent IC outcome. Notably, the latter patient had a dual pathology with hippocampal sclerosis despite the tumor’s location.

**Table 5:** Hippocampal resection in temporal lobe surgeries with neuroglial tumors.

Predictor		Hippocampectomy			
		12 months		Last FU	
Engel I vs. Engel II-III-IV	*p*-value	0.37^a^		0.59^a^	
Engel I-II vs. Engel III-IV	*p*-value	0.33^b^		0.34^b^	
		**With HP**	**Without HP**	**With HP**	**Without HP**
Outcomes (Engel)	IA	76.32 (29)	78.79 (26)	64.10 (25)	51.43 (18)
Percentage (%) and number of patients	IB, IC, ID	10.53 (4)	0 (0)	12.82 (5)	20 (7)
	II	10.53 (4)	12.12 (4)	20.51 (8)	20 (7)
	III	2.63 (1)	3.03 (1)	0 (0)	2.86 (1)
	IV	0 (0)	6.06 (2)	2.56 (1)	5.71 (2)

HP, Hippocampus. Results are displayed by *p*-values of Chi2-tests and Fisher’s exact tests comparing excellent with non-excellent (Engel Class I vs. Engel Classes II-IV); and favorable with poor outcomes (Engel Classes I-II vs. Engel Classes III-IV). Percentage and number of patients with respective outcomes are indicated accordingly. *P*-values from Chi2-test are indicated with ^a^, *p*-values from Fisher’s exact test are indicated with ^b^.

Altogether, we identified seven patients with coexistent hippocampal sclerosis in the observation period—six patients with GG, and one with DNET and hippocampal sclerosis. At the 12-month FU, six patients were completely seizure free and one had an Engel Class II outcome. At the longest FU (42.9 ± 18.5 months), five had Engel Class IA, one had Engel Class IC, and one had an Engel Class II outcome. These outcomes showed no significant differences compared to the outcomes of the group without hippocampal sclerosis but still undergoing hippocampal resection (*p* = 1.00, Fisher’s exact test, both for 12-month and longest FU, and both for comparing excellent outcomes with non-excellent outcomes, and favorable outcomes with poor outcomes).

When comparing the outcomes of temporal lobe surgeries on the dominant vs. non-dominant sides, the analysis did not reveal any significant differences (*p* = 0.90 and 0.82 by comparing excellent vs. non-excellent outcomes at the 12-month and longest FU, respectively, using the Chi2-test; and p = 1.00 and 1.00 when comparing favorable vs. poor outcomes at the 12-month and longest FU, using Fisher’s exact test).

## Discussion

In this retrospective study, we evaluated the 12-month and long-term postsurgical outcomes, specifically with regard to seizure control in 107 patients who had undergone epilepsy surgery for neuroglial tumors. A previous article from our center analyzed postsurgical 12-month seizure outcomes and neuropsychological outcomes in a partially overlapping patient population that underwent temporal lobe surgery (31). The present study aligns with previous studies, confirming that a complete lesion resection is an important prognostic factor which influences seizure control after surgery (14, 16, 32–37). This effect is likely more pronounced when taking into account that only patients with at least a 12-month FU were included in this study, and several patients who did not achieve satisfactory outcomes after the first surgery also underwent a second surgery, however, notably shorter than the 12-month FU, leading to their exclusion from the primary evaluation. For two of the four patients with this condition, an incomplete lesion resection was verified, and two of them achieved complete seizure freedom after the second surgery.

In this study, however, we could not identify additional factors which significantly and consistently contributed to the postsurgical outcomes. The history of bilateral tonic–clonic seizures, which was previously found to be a negative prognostic factor in some studies regarding GG (12, 15, 16), DNET (16, 38), or patients with long-term epilepsy-associated tumors (LEAT; 32), did not significantly influence postsurgical outcomes in our patient group. While some literature suggests differences in outcomes between GG and DNET [with GG displaying somewhat better outcomes than DNET (2, 14), and DNET slightly better than GG (31)], in our series we did not find significant differences either at the 12-month FU or at the longest possible FU. On the other hand, few studies directly compare outcomes in these distinct neuroglial tumor subtypes.

The impact of lesion localization is somewhat controversial in the literature: while a pediatric series, which also included gliomas, suggested that temporal lobe localization had a positive impact on outcomes (14), another study on LEATs, covering children and adults, associated temporal lobe localizations with worse outcomes (12).

A lower age at surgery and a shorter epilepsy duration were identified as positive predictors for good postsurgical outcomes in patients with GG (15, 37), in LEATs (32, 34), and in pediatric patients with temporomesial GG and DNET (39), whereas short epilepsy duration was found to be a good prognostic factor in GG (16, 33). In our series, however, we were unable to find convincing associations between age at surgery, epilepsy duration and postsurgical outcomes. A lower age at epilepsy onset appeared to be a significant positive predictor, but only when we compared favorable outcomes with poor outcomes.

Invasive diagnostics and side of the resection (dominant vs. non-dominant side) did not significantly impact the postsurgical outcomes in our patient cohort.

The necessity of resecting the ipsilateral amygdala and hippocampus in temporal lobe epilepsy associated with neuroglial tumors is controversial in the literature (16, 24, 25). In our series, there was no significant difference between those patients who underwent temporal lobe surgery with or without a hippocampectomy, either at 12 months or at the longest available FU. This also complies with previous results from our Department regarding 12-month outcomes on a partially overlapping patient population (31). However, this finding contradicts a pooled analysis involving several hundred patients, which identified the resection of the ipsilateral hippocampus as a positive predictor in temporal lobe epilepsy associated with neuroglial tumors (16). It’s worth noting that the named study included patients without pharmacorefractory epilepsy as well, and also some patients with shorter FUs (6 months), leading to higher rates of patients achieving Engel Class I outcomes. Moreover, at the longest available FU, we also observed a somewhat lower rate of cases with Engel Class IA and overall Engel Class I outcomes among patients without hippocampal resection. Overall, the larger sample size in the cited pooled patient group may also have contributed to the significant effects reported in the study (16). Additionally, the study does not report how many patients had temporo-mesial lesions or neuroglial tumors outside temporo-mesial structures in the temporal lobe.

In terms of determining the extent of resection (in addition to careful interpretation of findings from video EEG monitoring), the use of invasive diagnostics has notably declined in recent years, although this decrease was not statistically significant. Additionally, ECoG has been used under exceptionally rare circumstances in our center for the surgical treatment of neuroglial tumors. Surgical approaches have remained largely consistent throughout the study period. The primary goal is to achieve complete lesion resections, preferably with clear margins of healthy tissue of a few millimeters (supramarginal resection). In temporal lobe surgeries, particularly in cases of temporo-mesial or temporo-polar neuroglial tumors, most patients undergo amygdalohippocampectomy or temporal pole resection, or the combination of these two.

Our results have further clinical and practical implications. Firstly, we observed no difference in outcomes between patients who underwent hippocampal resection (primarily for temporo-mesial lesion localizations) and those who did not (primarily for temporo-lateral, temporo-polar and temporo-basal lesion localizations). It is also noteworthy that the majority of those patients with temporo-mesial neuroglial tumors who did not undergo hippocampal resection (with morphologically normal hippocampus on MRI) achieved excellent outcomes. This suggests that removing the hippocampus mechanistically together with temporo-mesial neuroglial tumors is not justified, unless the hippocampus shows structural alterations on the MRI. In a few cases, however, the hippocampus was also resected despite normal morphology; either based on the results of invasive diagnostics, or due to technical difficulties in dissecting the amygdala from the hippocampus (related to the close proximity of the lesion and hippocampus). In such instances, or when the hippocampus is only partially affected, a partial hippocampal resection (hippocampus head) can be discussed. Similarly, removing the amygdala together with the hippocampus, even in cases where the amygdala is not involved in the neuroglial tumor, is practical. In uncertain cases, additional invasive monitoring can provide further insight into the involvement of hippocampus in the seizure onset zone. Based on our results, in most cases there is no reason to extend resections to hippocampus in case of temporo-lateral, temporo-polar, or temporo-basal neuroglial tumors without temporo-mesial involvement. In general, a complete and supramarginal lesion resection should be pursued (in temporal as well as in extratemporal lesion localizations). Certainly, the results of neuropsychological examinations must be considered and the risk of possible cognitive deficits carefully evaluated, especially in those patients with lesions in the dominant hemisphere (31).

Our observational study has some limitations, including a single-center setting and retrospective data collection. The majority of neuroglial tumors in the evaluation were classified as GG, with DNET being less represented, which might pose challenges in drawing conclusions regarding the impact of histopathology. A similar challenge arises regarding lesion localizations, as most neuroglial tumors reside in the temporal lobe. The sample sizes for distinct extratemporal and multilobar localizations were too small to allow for separate analysis of these patient groups. Additionally, the number of patients with temporo-mesial lesions but without hippocampal resection was low, so respective results must be interpreted cautiously.

With the increasing popularity of stereotactic radiosurgery (40) and emerging new therapeutic modalities, particularly minimally invasive methods such as MRgLITT (41), additional studies are needed to compare these new techniques with conventional surgical methods. Another rapidly developing field involves the further molecular genetics classification of these lesions and the correlation of these genetic alterations with postsurgical outcomes. In addition to the previously listed genetic correlates, chromosomal copy number aberrations have been reported in both GG and DNET (42). Mutations in IDH1, 1p/19q codeletions and 9q deletions have been frequently observed in DNET (6, 43). While the presence of BRAF V600E mutations can influence the age of epilepsy onset, it does not appear to impact postsurgical outcomes (44). There is also an occasional association between neuroglial tumors and FCDs (3, 4, 18, 23). Interestingly, the PI3K-pathway appears to be involved in both the formation of FCDs and neuroglial tumors (45), and enhanced nestin mRNA was observed in FCDs with balloon cells and GG (46). These findings suggest that neuroglial tumors and FCDs may share molecular genetics and electrophysiological features much more similar than previously considered. Further elucidation of these phenomena, advancements in molecular classification, as recently described for specific cases of GG (47), and their correlation with postsurgical outcomes in future studies, will undoubtedly enhance our comprehension of the diversity of these lesions.

## Conclusion

Patients with neuroglial tumors are excellent epilepsy surgical candidates. The main prognostic factor for determining seizure outcome is a complete lesion resection. Extensive surgeries involving the resection of the hippocampus in temporal lobe epilepsies with neuroglial tumors do not necessarily improve postsurgical outcomes.

## Data availability statement

The raw data supporting the conclusions of this article will be made available by the authors, without undue reservation.

## Ethics statement

The studies involving humans were approved by Ethikkommission an der Medizinischen Fakultät der Rheinischen Friedrich-Wilhelms-Universität Bonn. The studies were conducted in accordance with the local legislation and institutional requirements. The ethics committee/institutional review board waived the requirement of written informed consent for participation from the participants or the participants’ legal guardians/next of kin because the study was a retrospective non-interventional study on already existing clinical data without any potential harmful risk. On the other hand, an informed consent was obtained for the phone interview, as regulated by the respective ethics committee.

## Author contributions

AR: Conceptualization, Data curation, Formal analysis, Writing – original draft, Writing – review & editing, Project administration, Visualization. PM: Data curation, Formal analysis, Visualization, Writing – original draft, Writing – review & editing. AB: Data curation, Formal analysis, Writing – original draft, Writing – review & editing, Visualization. NH: Data curation, Writing – review & editing, Formal analysis. TR: Data curation, Formal analysis, Writing – review & editing. VB: Data curation, Writing – review & editing, Formal analysis. HV: Data curation, Supervision, Writing – review & editing. RS: Data curation, Supervision, Writing – review & editing. CE: Conceptualization, Formal analysis, Supervision, Writing – original draft, Writing – review & editing.
